# Development and Evaluation of the Immunogenic Potential of an Unmodified Nucleoside mRNA Vaccine for Herpes Zoster

**DOI:** 10.3390/vaccines13010068

**Published:** 2025-01-13

**Authors:** Shun Zhang, Xiaojie Wang, Tongyi Zhao, Chen Yang, Lulu Huang

**Affiliations:** 1 Ningbo No. 2 Hospital, Guoke Ningbo Life Science and Health Industry Research Institute, Ningbo 315099, China; zhangshun@ucas.ac.cn; 2 Department of Clinical Pharmacy, School of Pharmaceutical Sciences, Shandong University, Jinan 250012, China; 2024211277@mail.sdu.edu.cn; 3 Technology Center, Shandong Freda Pharmaceutical Group, Jinan 250101, China; 4 Vaccine Center, School of Basic Medicine and Clinical Pharmacy, China Pharmaceutical University, Nanjing 211198, China; 3221091981@stu.cpu.edu.cn; 5 School of Life Science, Hangzhou Institute for Advanced Study, University of Chinese Academy of Sciences, Hangzhou 310024, China

**Keywords:** varicella-zoster virus, codon optimization, unmodified mRNA vaccine, Shingrix, immunogenicity

## Abstract

Background/Objectives: Approved mRNA vaccines commonly use sequences modified with pseudouridine to enhance translation efficiency and mRNA stability. However, this modification can result in ribosomal frameshifts, reduced immunogenicity, and higher production costs. This study aimed to explore the potential of unmodified mRNA sequences for varicella-zoster virus (VZV) and evaluate whether codon optimization could overcome the limitations of pseudouridine modification. Methods: We utilized artificial intelligence (AI) to design several unmodified gE mRNA sequences for VZV, considering factors such as codon preference and secondary structure. The optimized mRNA sequences were assessed for protein expression levels in vitro and were subsequently used to develop a vaccine, named Vac07, encapsulated in a lipid nanoparticle (LNP) delivery system. The immunogenicity of Vac07 was evaluated in mice. Results: Codon-optimized mRNA sequences showed significantly higher protein expression levels in vitro compared to wild-type (WT) sequences. Vaccination with Vac07 demonstrated immunogenicity in mice that was comparable to, or even superior to, the licensed Shingrix vaccine, characterized by a stronger Th1-biased antibody response and a slightly more robust Th1-type cellular response. Conclusions: Codon-optimized unmodified mRNA sequences may also represent a viable approach for mRNA vaccine development. These optimized sequences have the potential to lower production costs while possibly enhancing the immunogenicity of mRNA vaccines. Vac07, developed using this method, shows promise as a potentially more efficient and cost-effective mRNA vaccine candidate for VZV.

## 1. Introduction

Messenger RNA (mRNA) vaccines, as a novel biotechnological development, provide clear advantages in combating emerging, unknown pathogens as well as highly virulent and pathogenic agents [1,2]. These benefits include a relatively fast research and development process and the ability to generate robust immune responses that effectively balance both cellular and humoral immunity [3,4]. Immune receptors, such as TLR7/8, can recognize mRNA and directly activate the innate immune system, leading to the production of type I interferons [4,5,6]. Thus, while mRNA sequences serve as protein expression templates, they can also play the role of adjuvants. However, the excessive production of type I interferons may inhibit mRNA translation through various mechanisms, resulting in decreased protein expression and subsequently affecting the vaccine’s immunogenicity [5,7].

Research has shown that mRNA sequences modified with pseudouridine significantly enhance translation efficiency and stability [8,9]. Consequently, this modification strategy has been widely adopted by most mRNA pharmaceutical companies. However, despite the marked increase in translation efficiency associated with pseudouridine modification, its ability to activate the innate immune system is significantly reduced, which may negatively impact the overall immunogenicity of the vaccine [10]. Recent studies have also indicated that pseudouridine modification might cause ribosomal frameshifting, leading to off-target immune responses, an adverse effect that warrants careful consideration [11]. In terms of cost, pseudouridine is approximately ten times more expensive than regular uridine, resulting in higher expenses during the large-scale production of mRNA vaccines. However, as research into mRNA structure and function deepens, coupled with the rapid advancements in artificial intelligence technologies, new avenues and methodologies for developing optimized mRNA vaccines are emerging [12,13,14]. In this context, from the perspective of codon optimization, we modified the structural stability of the unmodified mRNA sequence to enhance its half-life. This is aimed at enhancing protein translation efficiency and immunogenicity and attempting to avoid the pseudouridine modification strategy.

In the field of global public health, the varicella-zoster virus (VZV) has garnered significant attention as an important pathogen. Shingles, a disease caused by the reactivation of the varicella virus, typically manifests as painful, localized blisters on the skin, inflicting considerable suffering and economic burden on patients [15]. The body’s defense against VZV infection primarily relies on T cell responses [16,17,18]. The currently available VZV vaccine, Shingrix, has been shown to significantly activate the body’s CD4^+^ T cell immune response, offering strong protection [19]. Additionally, RNA molecules possess an innate adjuvant effect, mainly through the induction of type I interferon production, which can trigger a robust cellular immune response [20,21]. As a result, the mRNA technology platform presents a theoretical advantage in the development of VZV vaccines.

Currently, the development of shingles vaccines focuses on glycoprotein E (gE) as the primary antigen, as it can induce a robust immune response in the body, including both cellular and humoral immunity, which is crucial for preventing viral infection and recurrence [22,23,24]. Therefore, we have also used the gE protein as the antigen for the development and research of the shingles mRNA vaccine.

This study aimed to design various gE mRNA sequences by taking into account key factors such as the mRNA secondary structure and codon bias, while leveraging the advantages of AI technology [12,14]. Through in vitro expression experiments, we identified the optimal candidate sequence and developed it into a VZV mRNA vaccine, followed by an immunogenicity evaluation in a mouse model. We are pleased to report that the unmodified VZV mRNA vaccine demonstrated immune responses comparable to, and in some cases even superior to, the positive control Shingrix. It induced a stronger Th-1 biased antibody response than Shingrix, as well as enhanced T cell responses. We hope that our research provides new insights for the development of mRNA vaccines for shingles, and that future efforts will focus on improving vaccine safety and efficacy while reducing production costs.

## 2. Materials and Methods

### 2.1. Ethics, Animals, and Immunization

All animal experiments were carried out in accordance with the Guidelines for the Care and Use of Laboratory Animals. C57BL/6 female mice, aged 6 weeks, were purchased from Hangzhou Ziyuan Laboratory Animal Co., Ltd., Hangzhou, China. and randomly grouped. In the immunogenicity experiment, the mRNA vaccine was administered intramuscularly at doses of 1 μg, 5 μg, or 10 μg. Additionally, Shingrix at 0.1 times the human dose was used as the positive control, and PBS was used as the negative control. Mouse serum was collected at a specified time for antibody detection. The mice were sacrificed at the specified time, and spleens and lymph nodes were collected for flow cytometry or ELISpot detection.

### 2.2. Sequence Design of mRNA

We designed nine additional sequences based on the wild-type VZV Oka strain (GenBank: AY253715.1), which served as Seq-01. Seq-02 to Seq-05 were designed using publicly available algorithms from Novopro, Genscript, GeneWiz, and JCAT, while Seq-06 to Seq-10 were designed based on the previously published LinearDesign algorithm [12]. The LinearDesign algorithm optimizes the mRNA sequence design by combining deterministic finite automaton (DFA) representation, dynamic programming for secondary structure prediction, and beam search to balance mRNA stability and codon adaptation efficiently.

### 2.3. mRNA Vaccine Preparation

The VZV mRNA vaccine (Vac07) was prepared according to a method established previously [25]. Briefly, T7 polymerase was used for in vitro mRNA transcription (SYNTHGENE), and Cap1-like analog reagents (SYNTHGENE) were added for capping. Then, the mRNA was purified using a Monarch RNA purification column (NEB, Ipswich, MA, USA) to obtain the mRNA molecule. For the preparation of LNP, briefly, an ionizable lipid, DSPC, cholesterol, and PEG were dissolved in ethanol at a molar ratio of 50:10:38.5:1.5 (all lipids were purchased from Sinopeg, Xiamen, China). The ionizable lipid was FS01, designed by Firestone Biotechnologies. The mRNA was dissolved in citrate buffer (pH 4.0). The lipid mixture and the mRNA solution were mixed at a volume ratio of 3:1 and prepared using microfluidics (INanoTML from Micro&Nano Biologics, Shanghai, China), with a total flow rate set at 12 mL/min. The prepared mRNA vaccine was dissolved in PBS and ultra-filtered using a 50 kDa Amicon ultracentrifuge filter (Merk, Darmstadt, Germany).

### 2.4. Measurement of Protein Expression in Vitro

Human embryonic kidney (HEK) 293T cells and DC 2.4 cells were purchased from Qingqi Biotechnology Development Co., Ltd., Shanghai, China. Both cell lines were tested and confirmed to be free of mycoplasma and other exogenous contaminants. Cells were cultured in high-glucose DMEM (BIOIND, Beersheba, Israel) supplemented with 10% FBS (BIOIND, Beersheba, Israel) and 1% penicillin–streptomycin (NCM Biotech, Suzhou, China). gE-mRNA was transfected into the cells using jetMESSENGER (Polyplus-transfection^®^, Göttingen, Germany). After 24 h, the cells were collected and incubated with anti-gE monoclonal antibody (1:200 dilution, Abcam) and PE-labeled anti-human IgG Fc (1:200 dilution, Biolgend, San Diego, CA, USA) successively for staining. Then, the Attune NxT flow cytometer (Thermo, Waltham, MA, USA) was used for detection, and the data were analyzed with FlowJo V.10.1 software (Tree Star, Ashland, OR, USA).

### 2.5. Measurement of gE-Specific IgG and IgG Subclasses

In a 96-well microplate (Greiner Bio-One, Friedrichsdorf, Germany), 50 ng of gE protein (Acro Biosystems, Beijing, China) was added to each well and incubated overnight at 4 °C. The next day, the plate was blocked with 2% bovine serum albumin (BSA) at room temperature. Then, the serum samples were diluted proportionally and added to the microplate for incubation at room temperature for 2 h. Subsequently, HRP-labeled goat anti-mouse IgG (1:50,000, Abcam, Cambridge, UK), IgG1 (1:5000, Southern Biotech, Birmingham, AL, USA), or IgG2c (1:5000, Southern Biotech, Birmingham, AL, USA) was used for incubation at room temperature. With the use of TMB substrate for signal development, the absorbance at a wavelength of 450 nm was measured. The dilution factor at which the OD value was greater than 4.1 times the background value was taken as the endpoint titer.

### 2.6. Analysis of Memory B Cell (MBC) Response

The biotinylated gE protein (Acro Biosystems, Beijing, China) was conjugated with fluorescent dyes BV421 or APC-streptavidin (Biolegend, San Diego, CA, USA) to form specific gE probes. After preparing single-cell suspensions from mouse spleen or lymph nodes, the cells were incubated with the gE probes. The cells were then stained with a LIVE/DEAD™ Fixable Aqua Dead Cell Stain Kit (Thermo, Waltham, MA, USA), followed by incubation with an antibody mixture. All incubation steps were performed at 4 °C. Finally, flow cytometry analysis was performed using the BD FACSymphony A3 (BD Biosciences, Franklin Lakes, NJ, USA), and data were analyzed with FlowJo V.10.1 software (Tree Star, Ashland, OR, USA). A list of the antibodies used is provided in Table S1.

### 2.7. Antigen Recall T Cell Assay

Two million mouse spleen cells were added to a 96-well U-bottom plate and incubated with gE protein (2 μg/mL, Acro Biosystems, Beijing, China) both in the presence and absence of Brefeldin A (BFA, Biolegend, San Diego, CA, USA) in the medium at 37 °C for 8 h. The cells were stained with the LIVE/DEAD™ Fixable Aqua Dead Cell Stain Kit (Thermo, Waltham, MA, USA) and then incubated with a mixture of cell surface marker antibodies and Fc receptor blocking reagent (Miltenyi, Cologne, Germany) for staining. Next, the cells were permeabilized using a Fixation/Permeabilization Solution Kit (BD Biosciences, Franklin Lakes, NJ, USA), followed by intracellular cytokine staining. Finally, flow cytometry analysis was performed using BD FACSymphony A3 (BD Biosciences, Franklin Lakes, NJ, USA), and the data were analyzed using FlowJo V.10.1 software (Tree Star, Ashland, OR, USA). A list of the antibodies used is shown in Table S1.

### 2.8. Enzyme-Linked Immunospot (ELISpot) Assay

The frequency of T cells specifically releasing IFN-γ or IL-2 was determined using a commercial ELISpot kit (Mabtech, Cincinnati, OH, USA) according to the provided instructions. Briefly, two million mouse spleen cells were added to the 96-well plate in the kit and incubated with complete medium containing or not containing gE protein (1 μg/mL, Acro Biosystems, Beijing, China) for 20 h. Subsequently, the corresponding antibodies in the kit were added for incubation, and the spots were counted with a CTL-Immunospot S6 analyzer. The results are expressed as the number of spot-forming cells (SFCs) per million stimulated cells.

### 2.9. Measurement of Antigen-Specific AIM^+^ T Cells and Tfh Cells

After two million mouse spleen cells were incubated with complete medium containing or not containing gE protein (2 μg per sample, Acro Biosystems, Beijing, China) for 20 h, the cells were incubated with the antibody mixture and Fc receptor blocking reagent (Miltenyi, Cologne, Germany) at room temperature for 20 min. Finally, flow cytometry analysis was performed using BD FACSymphony A3 (BD Biosciences, Franklin Lakes, NJ, USA), and the data were analyzed using FlowJo V.10.1 software (Tree Star). A list of the antibodies used is shown in Table S1.

### 2.10. Statistical Analysis

All statistical analyses were performed using GraphPad Prism v6.0 software. One-way analysis of variance (ANOVA) was used for comparisons between groups. *p*-values less than 0.05 were considered statistically significant (* *p* ≤ 0.05, ** *p* ≤ 0.01, *** *p* ≤ 0.001, **** *p* ≤ 0.0001).

## 3. Results

### 3.1. Sequence Design and in Vitro Translation Evaluation of Different gE mRNA

**Figure 1:**
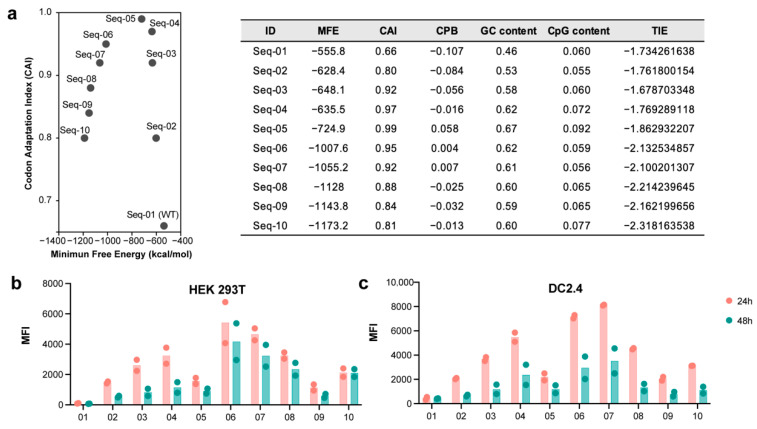
Design and translation of mRNAs into VZV gE protein. (**a**) mRNA sequences with different designs are shown, along with their respective CAI and MFE values. Additional relevant parameters are listed on the right. (**b**,**c**) gE mRNAs were transfected into HEK-293T or DC2.4 cells using jetMessenger. Cells were collected at 24 or 48 h post transfection and analyzed for gE antigen expression by flow cytometry. Data from two independent experiments are shown. CPB, codon pair bias; TIE, translation initiation efficiency.

We selected gE as the target antigen, designed to encode the full-length amino acid sequence of the gE gene from the Oka strain (GenBank: AY253715.1) [25]. Various strategies exist to improve protein expression, including codon optimization [26,27], the substitution of rare codons, increasing GC content [28], and optimizing secondary structures [12,13]. Using algorithms like LinearDesign, Genwiz, and Genscript, we designed optimal codon combinations for the mRNA sequences, resulting in a total of nine designed sequences [12]. Seq-01 represents the WT gE mRNA sequence, and Seq-02 to Seq-05 were designed using algorithms from Novopro, Genscript, GeneWiz, and JCAT, respectively. These design strategies significantly increased the Codon Adaptation Index (CAI) value [29] of the sequence (Figure 1a). In contrast, Seq-06 to Seq-10 were optimized using the LinearDesign algorithm [12], which combines deterministic finite automaton (DFA) representation, dynamic programming, and beam search to optimize the mRNA sequence design, aiming to improve both the CAI and stability (Minimum Free Energy, MFE) [30]. DFA efficiently compresses the search space, dynamic programming calculates the lowest free-energy secondary structure, and beam search accelerates the computation process, improving the efficiency of large-scale sequence designs. Therefore, the sequences designed with LinearDesign exhibit higher CAI values and lower MFE values. Additionally, we listed other parameters related to the sequences (Figure 1a). We performed in vitro transcription (IVT) to prepare ten mRNA sequences. One of the mRNA sequences was evaluated for purity using nucleic acid electrophoresis. The loading amounts were 80 ng, 40 ng, 20 ng, and 10 ng, revealing a single band in the RNA electrophoresis, confirming good purity (Figure S1a). The ten unmodified mRNAs were transfected into DC2.4 cells and HEK-293T cells, respectively. After 24 and 48 h, it could be seen that the optimized sequences were abundantly expressed, and the overall improvement of the mRNA sequence completed by the LinearDesign algorithm was relatively significant (Figure 1b,c). Notably, regardless of whether measured at 24 or 48 h, Seq-07 exhibited the highest protein expression in DC2.4 cells (Figure 1c). Furthermore, Seq-07 demonstrated a certain dose-dependent expression pattern following transfection (Figure S1b). Based on the comprehensive data collected, we ultimately selected Seq-07 as the VZV-mRNA vaccine sequence.

### 3.2. Humoral Immune Response Evoked in C57BL/6 Mice by Vac07

**Figure 2:**
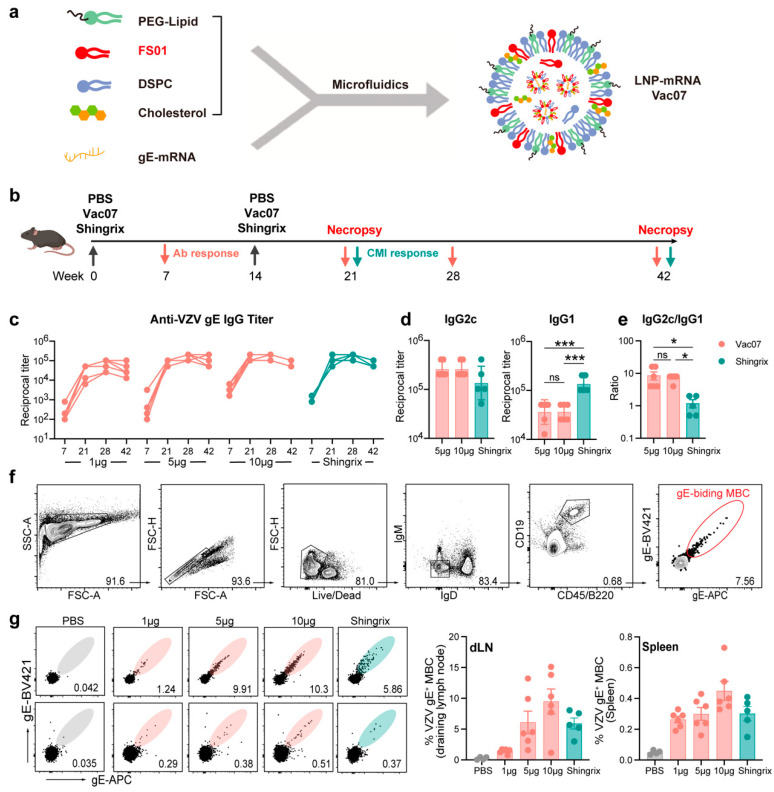
Evaluation of antibody and memory B cell responses induced by Vac07 and Shingrix. (**a**) Schematic representation of the formulation procedure for the VZV mRNA vaccine. (**b**) Experimental design: C57BL/6 mice were immunized i.m. with escalating doses of the VZV mRNA vaccine Vac07 (n = 6), 0.1 human dose of Shingrix (n = 5), or PBS (n = 4). Blood samples were collected at specified time points to measure antibody levels, while spleens and dLNs were harvested 28 days post booster for further analysis. (**c**) Anti-gE IgG titers were measured by ELISA, and the endpoint titers are shown. (**d**) Anti-gE IgG1 and IgG2c titers at day 28 were measured by ELISA, and the endpoint titers are shown. (**e**) The ratio of IgG2c/IgG1 is shown. (**f**) Gating strategy used for identifying class-switched gE-binding MBC. Data from one representative animal is shown. The gating strategy used was as follows: First, 1 million cells were stained, and IgM and IgD antibodies were used to exclude cells that had not undergone antibody class-switching. CD19 and CD45/B220 were then used to label memory B cells. Finally, gE protein conjugated with two fluorophores (APC and BV421) was used to label the class-switched memory B cells specific to gE binding. The numbers in the figure represent the percentage of each cell population relative to the parent gating step. The right panel shows the data summary results. (**g**) Frequencies of class-switched (IgD^-^IgM^-^) gE-specific MBCs in dLNs and spleens were analyzed by flow cytometry. The left panel shows the flow cytometry representative plots, with each plot displaying a cell count of 3000. The numbers in the figure represent the frequency of class-switched gE-binding MBCs. Data are shown as mean ± SEM. One-way ANOVA with multiple comparisons tests was used for the analysis of statistical significance. ns, indicating no significant difference; * *p* ≤ 0.05, *** *p* ≤ 0.001.

Utilizing the novel ionizable cationic lipid FS01 in conjunction with other lipids, we employed microfluidics to formulate an mRNA vaccine, designated as Vac07 (Figure 2a). The immunogenicity of Vac07 and Shingrix was assessed and compared in C57BL/6 mice that were intramuscularly vaccinated with increasing doses of Vac07 (1 μg, 5 μg, 10 μg) or with a 0.1 human dose of Shingrix on day 0 and day 14 (Figure 2b).

After vaccination, we measured the body weight of the mice weekly. Mice vaccinated with Vac07 showed a steady increase in body weight, which initially suggests good safety (Figure S2). In terms of antibody response, two doses of Vac07 induced a high level of gE-specific IgG in a dose-dependent manner. Mice that received a moderate dose (5 μg) or high dose (10 μg) of the mRNA vaccine exhibited a more robust antibody response, which was comparable to the antibody levels induced by Shingrix (Figure 2c). We further analyzed the gE-specific IgG subclasses and found that Vac07 was more effective at inducing a Th1-biased IgG response, as evidenced by the induction of higher IgG2c and lower IgG1 titers compared to Shingrix, resulting in a higher IgG2c/IgG1 ratio in the Vac07 group (Figure 2d, e). Additionally, four weeks after booster immunization, we measured the frequency of class-switched IgD⁻IgM⁻ memory B cells (MBCs) specific to the gE antigen in the spleen and draining lymph nodes (dLNs), which were analyzed according to a predefined gating strategy (Figure 2f). The 10 μg Vac07 induced slightly higher levels of gE⁺ MBCs in both anatomical lymphoid organs compared to Shingrix (Figure 2g). This suggests that, compared to Shingrix, Vac07 induces a comparable or potentially stronger humoral immune response.

### 3.3. Cell-Mediated Immune Response Elicited in C57BL/6 Mice by Vac07

**Figure 3:**
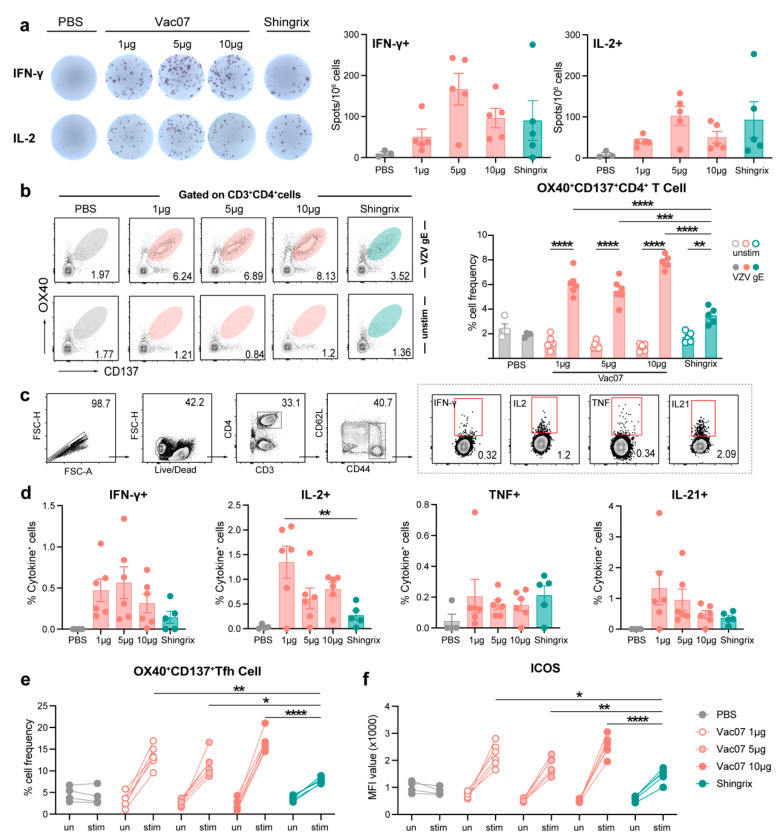
Evaluation of T cell responses induced by Vac07 and Shingrix. C57BL/6 mice were i.m. immunized with escalating doses of Vac07 or 0.1 human dose of Shingrix on day 0 and day 14. Spleens were harvested 7 days (**a**) or 28 days (**b**–**f**) after the boost. (**a**) Splenocytes were stimulated with 2 μg/mL gE antigen for 20 h. Frequencies of IFN-γ- or IL-2-secreting T cells were assessed by ELISpot. (**b**) Splenocytes were stimulated with or without 2 μg/mL of gE antigen for 20 h. The frequency of AIM^+^CD4^+^ T cells was measured by flow cytometry. The left panel shows the flow cytometry representative plots, with each plot displaying a cell count of 20,000. The numbers in the figure represent the frequency of AIM^+^ CD4^+^ T cells. The right panel shows the data summary results. (**c**) Splenocytes were stimulated with or without 2 μg/mL gE antigen for 8 h in the presence of Brefeldin A. The gating strategy used to analyze IFN-γ-, IL-2-, TNF-, or IL-21-producing CD4^+^ T cells in the spleens of mice is shown. Data from one representative animal are presented. The gating strategy used was as follows: First, 1 million cells were stained, and CD4^+^ T cells were identified using CD3 and CD4 antibodies. CD44 and CD62L antibodies were then used to label memory T cells. Finally, cytokine-specific memory T cells were identified using four cytokine antibodies (IFN-γ, IL-2, TNF, IL-21). The numbers in the figure represent the percentage of each cell population relative to the parent gating step. The right panel shows the data summary results. (**d**) Frequencies of IFN-γ-, IL-2-, TNF-, or IL-21-secreting CD4^+^ T cells were determined by flow cytometry. (**e**) AIM^+^ Tfh cells were determined by flow cytometry. (f) The ICOS expression on OX40^+^CD137^+^CXCR5^+^CD4^+^ Tfh cells was evaluated. MFI value is shown. Data represent mean ± SEM. One-way ANOVA with multiple comparisons tests was used for the analysis of statistical significance. * *p* ≤ 0.05, ** *p* ≤ 0.01, *** *p* ≤ 0.001.**** *p* ≤ 0.0001.

Given that T cell responses play a key role in preventing VZV reactivation [16,31], we focused more on the T cell-mediated responses elicited. First, on day 7 after the booster, spleens were harvested, and ELISpot assays revealed that the 5 µg Vac07 induced the highest levels of IFN-γ- and IL-2-secreting T cells (Figure 3a). On day 28 post booster, Vac07-vaccinated mice showed a stronger induction of gE-specific AIM^+^ (activation-induced markers OX40 and CD137) CD4^+^ T cells upon antigen stimulation in spleens (Figure 3b). We further employed a well-established intracellular cytokine recall assay (Figure 3c) to assess the induction of gE-specific CD4^+^ T cells producing IFN-γ, TNF, IL-2, and IL-21 following gE antigen stimulation (Figure 3d). Compared with Shingrix, Vac07 could moderately increase the frequency of Th1-type CD4^+^ T cells that specifically secrete IL-2 and IFN-γ in the spleen, while the frequency of TNF-secreting CD4^+^ T cells was basically the same. Moreover, there was no apparent dose-dependent effect. IL-21, a cytokine specifically secreted by T follicular helper (Tfh) cells, was then examined. We observed a significant expansion of gE-specific AIM^+^ Tfh cells, defined as OX40^+^CD137^+^CD4^+^CXCR5^+^ T cells, particularly in mice vaccinated with Vac07 (Figure 3e). Additionally, these vaccine-induced Tfh cells displayed a highly activated phenotype with elevated ICOS expression (Figure 3f). Collectively, these findings demonstrate that Vac07 induces VZV-specific T cell responses in mice that are comparable to, or even slightly stronger than, those induced by Shingrix.

## 4. Discussion

Although mRNA vaccines have made rapid progress, they are limited by their chemical instability, which makes them prone to degradation. Consequently, the storage and distribution of mRNA require cold chain technologies, thereby restricting the widespread use of these vaccines in developing countries [32]. Furthermore, the inherent instability of mRNA in vivo, along with its susceptibility to recognition and clearance by the innate immune system, can lead to low protein expression efficiency and reduced immunogenicity [5,33]. While the translation efficiency of mRNA modified with pseudouridine is significantly enhanced, this modification also diminishes the natural immune activation capability, consequently lowering its immunogenicity [10].

This study focused on the development of a promising unmodified VZV mRNA vaccine, Vac07, through the codon optimization of the gE mRNA. Comprehensive in vitro and in vivo evaluations were conducted to assess its potential. In mRNA sequence design, we carefully considered factors such as secondary structure and codon usage instead of pseudouridine modification. This approach ensures that translation efficiency and stability are optimized within a certain range, while also promoting the activation of innate immunity. A well-balanced innate immune response is crucial and must be carefully controlled to an appropriate level in order to generate high-quality adaptive vaccine responses while ensuring safety. Uridine, compared to pseudouridine, induces a stronger innate immune response, and excessive immune system stimulation may lead to negative effects, such as an increased risk of inflammation [10,34,35]. We also acknowledge that it is not possible to completely eliminate the risk of such effects. In this study, we propose an approach where the gE mRNA sequence is designed based on a linear design to lower the MFE, improve the structural compactness of the mRNA molecules, and enhance mRNA stability. More stable mRNA can remain in cells for a longer period, thereby reducing unnecessary immune responses. However, we cannot rule out the possibility that unmodified mRNA vaccines may perform less effectively compared to pseudouridine-modified mRNA, given the widespread use of pseudouridine modification in mRNA vaccines. According to the experimental results in this study, the mRNA vaccine designed using linear design showed effective expression both in vitro and in vivo, and in comparison with the positive control Shingrix, which is the most widely used and effective vaccine on the market, it activated an immune response that was comparable to or even stronger than that of Shingrix. Furthermore, the mice’s weight remained within the normal range following vaccination. Future studies should include a head-to-head comparison between unmodified and pseudouridine-modified mRNA vaccines. Additionally, further evaluation of the safety of this vaccine is still warranted.

Therefore, the codon optimization technique used in this study may potentially serve as a way to avoid pseudouridine modification, with the potential to reduce vaccine production costs. However, to translate these findings into clinical applications, further extensive and detailed work is needed to explore the underlying mechanisms of action, as well as to conduct thorough safety evaluations. Future research should continue to investigate the complex biological behavior of unmodified mRNA sequences in vivo, refine vaccine manufacturing processes, and ultimately provide a more efficient, safe, and cost-effective vaccine option for the global prevention and control of varicella-zoster virus or other pathogens. In addition to their application in infectious disease vaccines, mRNA technology can also be utilized in the field of tumor therapy. Given that the tumor microenvironment is often immunosuppressive [36,37], novel unmodified mRNA drugs optimized through codon modification hold promise for overcoming this immunosuppressive environment and improving tumor treatment outcomes.

## 5. Conclusions

In conclusion, this study suggests that codon-optimized unmodified mRNA sequences could also serve as a potential direction for mRNA vaccine development. The resulting vaccine, Vac07, showed immunogenicity comparable to or even superior to the licensed Shingrix vaccine, with a robust Th1-biased antibody response and a slightly stronger Th1-type cellular response. Furthermore, the use of unmodified mRNA sequences may reduce production costs while maintaining or potentially improving vaccine efficacy.

## Data Availability

All data are available upon reasonable request to the corresponding authors.

## Supplementary Materials

The following supporting information can be downloaded at https://www.mdpi.com/article/10.3390/vaccines13010068/s1, Figure S1: Electrophoretic analysis of VZV gE-mRNA synthesized by IVT and its dose-dependent expression during trsnsfection; Figure S2: Monitoring of body weight following vaccination; Table S1: List of anti-mouse antibodies used for FACS analysis.
