# Supplementary material for: Development and Evaluation of the Immunogenic Potential of an Unmodified Nucleoside mRNA Vaccine for Herpes Zoster
Source: Vaccines (Basel). 2025 Jan 13;13(1):68. doi: 10.3390/vaccines13010068 (PMC11768781; doi:10.3390/vaccines13010068)
Supplement: Supplementary file 1 [file vaccines-13-00068-s001.zip › vaccines-3395289-supplementary.pdf]

Supplemental Figure S1

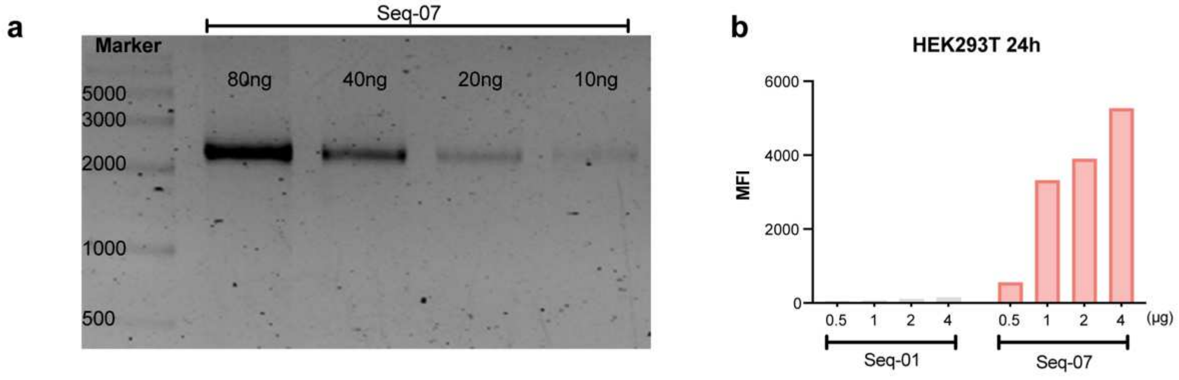

Figure. S1. Electrophoretic analysis of VZV gE-mRNA synthesized by IVT and its dose-dependent expression during trsnsfection.

(**a**)Representative electrophoresis results are shown. (**b**)The gE mRNAs Seq01 and Seq07 were transfected into HEK-293T at 0.5 μg, 1 μg, 2 μg and 4 μg. Cells were harvested after 24 hours and evaluated for gE antigen expression by flow cytometry.

Supplemental Figure S2

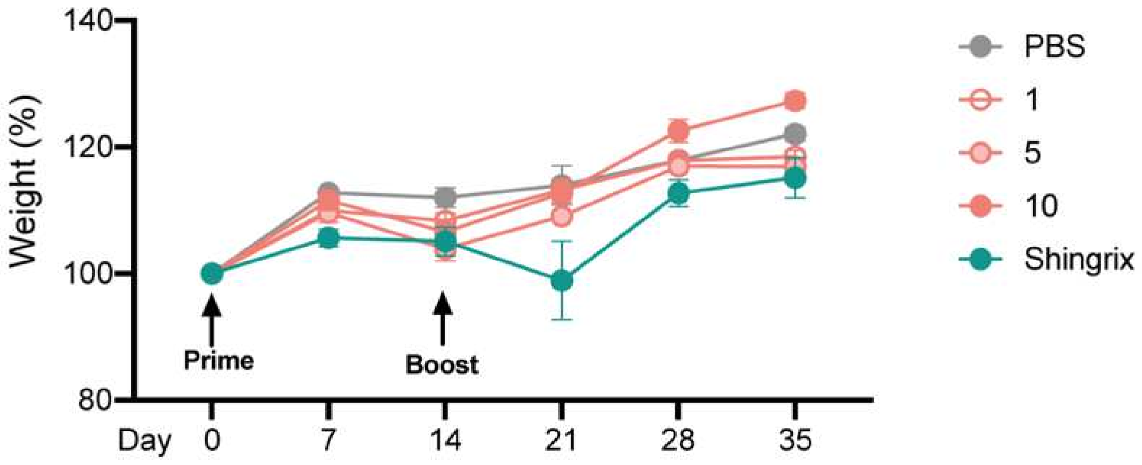

Figure. S2. Monitoring of body weight following vaccination.

C57BL/6 mice were i.m. immunized with escalating doses of Vac07 or 0.1 human dose of Shingrix on day 0 and day 14 with PBS-injected mice serving as controls. Body weight was monitored, and percentage of weight change is shown.

Supplemental Table S1

Table S1. List of anti-mouse antibodies used for FACS analysis

Antibody	Clone	Manufacturer
IgM	RMM-1	Biolegend
IgD	11-26c.2a	Biolegend
CD45R/B220	RA3-6B2	Biolegend
CD19	6D5	Biolegend
CD3	17A2	Biolegend
CD4	GK1.5	Biolegend
CD44	IM7	Biolegend
CD62L	MEL-14	Biolegend
IFN-γ	XMG1.2	Biolegend
TNF	MP6-XT22	Biolegend
IL-2	JES6-5H4	Biolegend
IL-21	mhalx21	Invitrogen
CD4	RM4-5	Biolegend
CXCR5	L138D7	Biolegend
PD1	29F.1A12	Biolegend
ICOS	C398.4A	Biolegend
OX40	OX-86	Biolegend
CD137	17B5	Biolegend
